# Lateral Medullary Infarction With Atonic Bladder and Lateropulsion

**DOI:** 10.7759/cureus.54492

**Published:** 2024-02-19

**Authors:** Koji Hayashi, Midori Ueda, Asuka Suzuki, Yuka Nakaya, Hina Hamada, Mamiko Sato, Kouji Hayashi, Yasutaka Kobayashi

**Affiliations:** 1 Department of Rehabilitation Medicine, Fukui General Hospital, Fukui, JPN; 2 Graduate School of Health Science, Fukui Health Science University, Fukui, JPN

**Keywords:** lateral medullary infarction, anuresis, atonic bladder, lateropulsion, lateral medullary syndrome (wallenberg syndrome)

## Abstract

Lateral medullary infarction (LMI), or Wallenberg syndrome, can develop various symptoms, but it is rare that ipsilateral axial lateropulsion (or body lateropulsion, BL) or atonic bladder (AB) are caused by LMI. This report describes a case of LMI with both BL and AB. A 77-year-old man, with a history of hypertension and diabetes, developed acute left BL and anuresis. A neurological exam showed right gaze nystagmus, slight dysarthria and dysphagia, right dysesthesia in the trunk, and ataxia in the left limbs and trunk. Horner’s syndrome and paralysis were unremarkable. Brain magnetic resonance imaging revealed hyperintensity in the lateral medulla oblongata. Cystometry revealed AB, although the patient had the urge to urinate. Owing to acute therapy, although trunk ataxia was presented for several months, BL and anuresis were recovered on day 15 and day 35, respectively. Here, we describe the potential mechanisms of BL and AB caused by LMI.

## Introduction

Lateral medullary infarction (LMI), or Wallenberg syndrome, is a clinical manifestation of infarction in the territory of the posterior inferior cerebellar artery and its branches [1,2]. Patients with LMI can develop various symptoms, including vertigo, nausea/vomiting, headache, ipsilateral Horner syndrome, skew deviation of eyes, nystagmus, dysphagia, dysarthria, hoarseness, ipsilateral diminished gag reflex, cerebellar ataxia, and crossed sensory disturbance (ipsilateral face, contralateral body) [1,2]. In addition, ipsilateral axial lateropulsion (or body lateropulsion, BL) may be rarely present in LMI [3-5]. Moreover, urinary symptoms caused by LMI are extremely rare. Here, we describe a case of LMI developing both LMI and atonic bladder (AB).

## Case presentation

A 77-year-old man, with a history of hypertension and diabetes, developed acute left BL and anuresis. A neurological exam showed right gaze nystagmus, slight dysarthria and dysphagia, right dysesthesia in the trunk, ataxia in the left limbs and trunk, and his body leaning to the left when walking. Horner’s syndrome and paralysis were unremarkable. Blood tests were unremarkable except for elevated brain natriuretic peptide, blood sugar, hemoglobin A1c, and D-dimer (Table 1).

**Table 1:** The result of blood tests on admission.

Inspection items	Result	Reference range	Inspection items	Result	Reference range
White blood cell count	5800/μl	(3300–8600)	Blood urea nitrogen	12.4 mg/dl	(8.0–20.0)
Red blood cell count	412×10⁴/μl	(386–492×10⁴)	Creatinine	0.54 mg/dl	(0.46–0.79)
Hemoglobin	13.0 g/dl	(11.6–33.4)	Natrium	137 mmol/l	(138–145)
Blood platelet	23.0×10⁴/μl	(15.8–34.8)	Potassium	3.7 mmol/l	(3.6–4.8)
Total protein	6.8 g/dl	(6.6–8.1)	Chlorine	101 mmol/l	(101–108)
Albumin	3.9 g/dl	(4.1–5.1)	C-reactive protein	0.01 mg/dl	(0.00–0.14)
Total bilirubin	0.7 mg/dl	(0.4–1.5)	Low-density lipoprotein cholesterol	128 mg/dl	(70–140)
Alkaline phosphatase	309 U/l	(106–322)	High-density lipoprotein cholesterol	88 mg/dl	(40–70)
Aspartate aminotransferase	17 U/l	(13–30)	Triglyceride	79 mg/dl	(30–149)
Alanine aminotransferase	11 U/l	(7–30)	Prothrombin time	11.2 sec	(10.0–13.0)
Lactate dehydrogenase	189 U/l	(124–222)	Prothrombin time-international normalized ratio	1.03	(0.85–1.15)
Creatine kinase	76 U/l	(60–230)	Activated partial thromboplastin time	26.4 sec	(25.0–40.0)
γ-glutamyl transpeptidase	17 IU/l	(<50)	Fibrinogen	245.9 mg/dl	(150-400)
Cholinesterase	327 U/l	(213–501)	Fibrin degradation products	4.4 μg/ml	(<5.0)
Glucose	295 mg/dl	(73–109)	D-dimer	2.3 μg/ml	(<1.0)
Hemoglobin A1c	7.7%	(<5.5%)	Brain natriuretic peptide	186.2 pg/ml	(<18.4)

**Figure 1:**
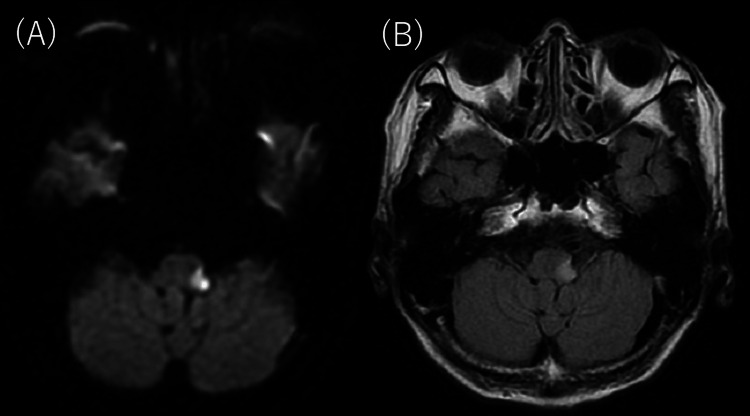
The results of brain magnetic resonance imaging (MRI). (A) Diffusion-weighted brain MRI showing hyperintensity in the left lateral medulla oblongata. (B) T2-weighted fluid-attenuated inversion recovery (T2-FLAIR) brain MRI showing hyperintensity in areas that were hyperintense on diffusion-weighted imaging.

**Figure 2:**
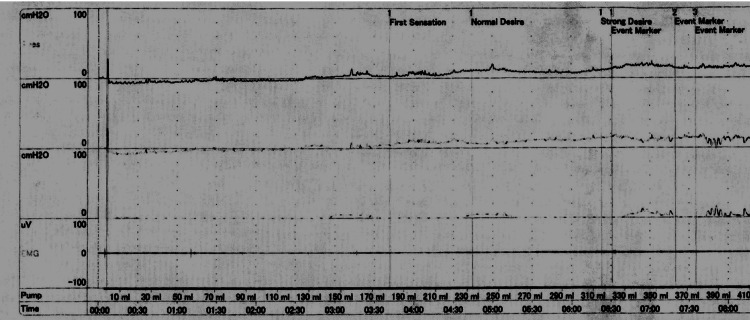
The result of cystometry. Cystometry showed that though the patient had an urge to urinate after injecting 180 ml of water, no contraction of the detrusor muscle was observed, although the intravesical pressure continued to rise slowly with the infusion.

Brain magnetic resonance imaging (MRI) revealed hyperintensity in the lateral medulla oblongata (Figure 1). Cystometry showed AB although the patient had the urge to urinate (Figure 2). Carotid ultrasound showed no plaque on the lumen of the bilateral carotid arteries. Transthoracic echocardiography revealed no cardiac hypertrophy, valvular heart disease, or wall motion abnormalities. Holter electrocardiogram did not reveal atrial fibrillation.

He was treated with argatroban, clopidogrel, edaravone, and rehabilitation therapy, including physical, occupational, and speech therapies. Although trunk ataxia was presented for several months, BL and anuresis were recovered on day 15 and day 35, respectively. He was discharged from our hospital with near independence in activity of daily living on day 49, although a trunk ataxia was slightly preserved.

## Discussion

To the best of our knowledge, this is the first report of LMI with both BL and anuresis. In addition to these findings, the patient developed common symptoms in LMI, including dysarthria, dysphagia, contralateral sensory disturbance, ipsilateral limb ataxia, and truncal ataxia. Since the onset of these symptoms was sudden, they were consistent with a vascular disorder. Brain MRI revealed typical LMI in the left. Based on these findings, the patient was a typical case of LMI, except for developing BL and AB.

BL by LMI is rare, but five possible pathways of BL are estimated in the literature: the dorsal spinocerebellar tract (DSCT), the descending lateral vestibulospinal tract (LVST), the vestibulo-thalamic pathway (ascending graviceptive pathway), the dentatorubrothalamic pathway, or the thalamocortical fascicle [6]. Among five pathways, DSCT and LVST may be involved in the infarction in the medulla oblongata [6]. Whereas the involvement of DSCT can cause ataxia, the involvement of LVST cannot [7]. In our case, because ataxia is caused, we estimated that DSCT may be involved by LMI.

On the other hand, as far as we know, two cases of LMI-developed dysuria have been reported in English (only abstract) [8,9]. One case is right LMI presenting with dysuria caused by detrusor-sphincter dyssynergia [8]. The other case is left LMI, presenting with dysuria caused by AB [9]. In our case, cystometry showed that though the patient had the urge to urinate after injecting 180 ml of water, no contraction of the detrusor muscle was observed, although the intravesical pressure continued to rise slowly with the infusion. This finding is diagnostic of AB, as well as the latter case.

**Figure 3:**
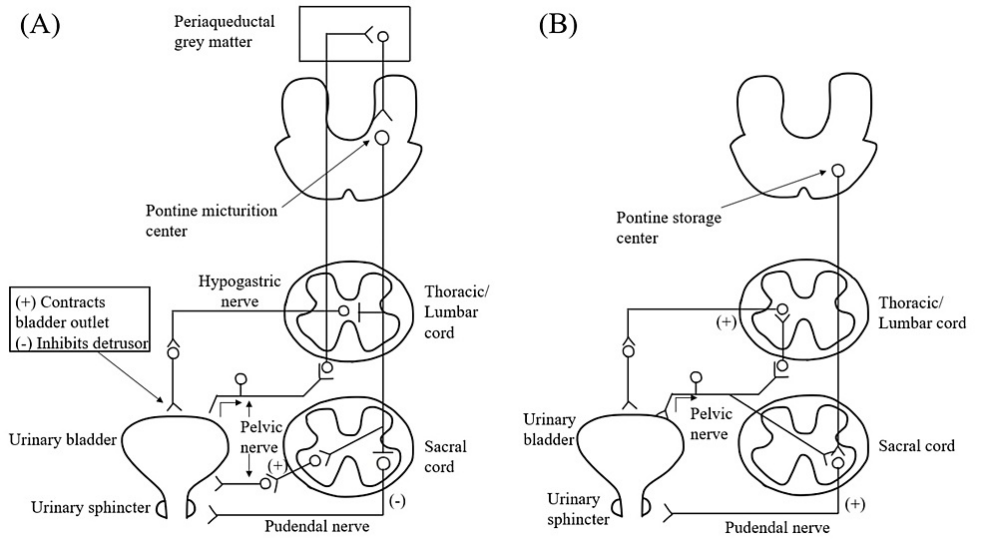
Schematic diagram of central nervous pathways for micturition (A) and urine storage (B). A higher urination center and urine storage center control the micturition and urine storage via sympathetic and parasympathetic nerves. (+) Contracts urinary bladder outlet; (-) inhibits detrusor.

In this paragraph, we discuss the reason for the AB by LMI. Figure 3 is a schematic diagram of the neural pathway that controls micturition (Figure 3A) and urine storage (Figure 3B) in humans.

The sympathetic efferent pathway of the hypogastric nerve, which originates from the thoracolumbar spinal cord, inhibits urination by relaxing the detrusor muscle of the bladder and contracting the bladder outflow tract [10]. The efferent path of the parasympathetic nerve, which is included in the pelvic nerve and originates from the sacral spinal cord, contracts the detrusor muscle [10]. Pelvic nerve afferents originate from the bladder, and increases in intravesical pressure are transmitted to the central nervous system via these afferents [10]. On the other hand, the sphincter muscle is controlled by the pudendal nerve that originates from the sacral spinal cord [10]. These mechanisms are controlled by the higher urination center and urine storage center [10]. During urination (Figure 3A), the descending path from the pontine micturition center located near the locus coeruleus in the pontine tegmentum suppresses the hypogastric nerve at the thoracolumbar spinal cord level and excites the pelvic nerve at the sacral spinal cord level [8,10]. It contracts the detrusor muscle and at the same time relaxes the sphincter by suppressing the pudendal nerve. On the other hand, during urine storage (Figure 3B), the descending path from the micturition inhibition area near the pontine micturition center excites the pudendal nerve, causing the sphincter to contract [8,10]. In our case, it is thought that the descending path from the pontine micturition center was damaged at the medulla oblongata level by LMI, leading to AB.

## Conclusions

As far as we know, this is the first report about LMI developing both BL and AB. Because the associated fibers of BL and AB pass through the medulla oblongata, both of them may be developed by LMI. Accumulation of a large number of cases is needed to reveal the underlying mechanism of developing AB in LMI.
